# Patients Contributing to Visit Notes: Mixed Methods Evaluation of OurNotes

**DOI:** 10.2196/29951

**Published:** 2021-11-08

**Authors:** Jan Walker, Suzanne Leveille, Gila Kriegel, Chen-Tan Lin, Stephen K Liu, Thomas H Payne, Kendall Harcourt, Zhiyong Dong, Patricia Fitzgerald, Matthew Germak, Lawrence Markson, Sara L Jackson, Hannah Shucard, Joann G Elmore, Tom Delbanco

**Affiliations:** 1 Division of General Medicine Beth Israel Deaconess Medical Center Boston, MA United States; 2 Harvard Medical School Boston, MA United States; 3 College of Nursing and Health Sciences University of Massachusetts Boston Boston, MA United States; 4 School of Medicine University of Colorado Aurora, MA United States; 5 General Internal Medicine Dartmouth-Hitchcock Medical Center Lebanon, MA United States; 6 Department of Medicine University of Washington School of Medicine Seattle, MA United States; 7 Primary Care Beth Israel Lahey Health Needham, MA United States; 8 Clinical Information Systems Beth Israel Deaconess Medical Center Boston, MA United States; 9 Department of Biostatistics University of Washington School of Medicine Seattle, MA United States; 10 David Geffen School of Medicine University of California, Los Angeles Los Angeles, MA United States

**Keywords:** electronic health record, previsit information, physician-patient relations, patient portal, mobile phone

## Abstract

**Background:**

Secure patient portals are widely available, and patients use them to view their electronic health records, including their clinical notes. We conducted experiments asking them to cogenerate notes with their clinicians, an intervention called *OurNotes.*

**Objective:**

This study aims to assess patient and provider experiences and attitudes after 12 months of a pilot intervention.

**Methods:**

Before scheduled primary care visits, patients were asked to submit a word-constrained, unstructured interval history and an agenda for what they would like to discuss at the visit. Using site-specific methods, their providers were invited to incorporate the submissions into notes documenting the visits. Sites served urban, suburban, and rural patients in primary care practices in 4 academic health centers in Boston (Massachusetts), Lebanon (New Hampshire), Denver (Colorado), and Seattle (Washington). Each practice offered electronic access to visit notes (open notes) to its patients for several years. A mixed methods evaluation used tracking data and electronic survey responses from patients and clinicians. Participants were 174 providers and 1962 patients who submitted at least 1 previsit form. We asked providers about the usefulness of the submissions, effects on workflow, and ideas for the future. We asked patients about difficulties and benefits of providing the requested information and ideas for future improvements.

**Results:**

Forms were submitted before 9.15% (5365/58,652) eligible visits, and 43.7% (76/174) providers and 26.76% (525/1962) patients responded to the postintervention evaluation surveys; 74 providers and 321 patients remembered receiving and completing the forms and answered the survey questions. Most clinicians thought interim patient histories (69/74, 93%) and patient agendas (72/74, 97%) as good ideas, 70% (52/74) usually or always incorporated them into visit notes, 54% (40/74) reported no change in visit length, and 35% (26/74) thought they saved time. Their most common suggestions related to improving notifications when patient forms were received, making it easier to find the form and insert it into the note, and educating patients about how best to prepare their submissions. Patient respondents were generally well educated, most found the history (259/321, 80.7%) and agenda (286/321, 89.1%) questions not difficult to answer; more than 92.2% (296/321) thought sending answers before the visit a good idea; 68.8% (221/321) thought the questions helped them prepare for the visit. Common suggestions by patients included learning to write better answers and wanting to know that their submissions were read by their clinicians. At the end of the pilot, all participating providers chose to continue the *OurNotes* previsit form, and sites considered expanding the intervention to more clinicians and adapting it for telemedicine visits.

**Conclusions:**

*OurNotes* interests patients, and providers experience it as a positive intervention. Participation by patients, care partners, clinicians, and electronic health record experts will facilitate further development.

## Introduction

### Background

Patient engagement is essential for improving patient experience, reducing the cost of care, and improving population health [1]. With the proliferation of secure patient portals over the past decade, patients have gained a powerful engagement tool [2,3]. Millions of patients and their care partners now go on the web to communicate with members of the care team and review their medical records. Beginning in April 2021, as part of the 21^st^ Century Cures Act, patients nationwide have access to virtually all information in their electronic health records, including the notes of their clinicians [4].

Hundreds of provider organizations implemented *open notes* on their patient portals following publication of the first open notes study in 2012 [5,6]. Patients rate note reading as very important for remembering what happened in their office visits, remembering their care plan, taking care of their health, and feeling in control of their care [7-10]. Patients have also expressed interest in doing more than passively reading their notes. In the first study of open notes, 60% wanted to be able to comment on notes, and 35% wanted to be able to approve their notes [5]. Though few primary care physicians thought patients should approve the content of notes, approximately one-third agreed they should be able to comment. Researchers have since shown that reading notes and inviting patients to comment on their notes can benefit patient safety [11-16]. Though many clinicians face time pressures, a majority report spending the same or less time writing open notes [17]. The National Academy of Medicine’s Vital Directions for Health and Health Care initiative recommends that patients and authorized family caregivers be able to modify their health records [18]. Though many ambulatory care practices collect information from patients in brief questionnaires, we know of no efforts inviting them to actively write in their records. As a step in this direction, we decided to mount a pilot inviting patients to contribute to their visit notes, thus transforming the official record of their care into cogenerated *OurNotes*.

We designed, implemented, and evaluated portal mechanisms for patients and their care partners to contribute to notes of providers documenting their ambulatory visits. Following advice from expert interviews, we determined to focus on previsit communications by asking patients to (1) write a brief history since the last visit and (2) to identify up to three priorities for the upcoming visit [19].

### Objectives

Our objectives were to have providers incorporate this previsit input into their visit notes, thereby taking a first step toward *OurNotes*, and to assess patient and provider experiences with this pilot intervention. We hypothesized that clinicians and patients would find it beneficial to submit the information before visits, especially patients with a heavy burden of illness, and that the intervention would be time-neutral or possibly save time for clinicians. This paper reports the principal findings from 12-month pilots of this intervention in 4 sites.

## Methods

### Overview

Primary care practices associated with 4 sites across the United States participated: Beth Israel Deaconess Medical Center (BIDMC) in Boston, UCHealth (UCH) in Aurora, Colorado (including a women’s health clinic), Dartmouth-Hitchcock Medical Center (DHMC) in Lebanon, New Hampshire, and University of Washington Medicine (UW) in Seattle (including a safety net clinic and a clinic that serves primarily privately insured patients). Each site has well-established mechanisms for offering open notes to patients. The *OurNotes* and site project teams developed an overall intervention design and evaluation plan. The study protocols were approved by the institutional review boards of the participating institutions.

### Intervention

The intervention included primary care providers and patients who were registered on the patient portal. During the 12-month pilot period, these patients were invited via a secure portal message or email to complete a form addressing two questions before a scheduled visit: *How have you been since your last visit?* *What are the most important things you would like to discuss at your visit? (list up to 3)*. Answers were limited to 2000 and 300 characters, respectively. Clinicians could view the completed forms before and during the visit, and when they wrote the visit note, they could decide whether and to what degree to incorporate the patient’s words.

Between mid-2017 and early 2019, each site built its own implementation process and supported the information technology infrastructure. Although all asked the same two questions up to 1 week before the visit, the sites differed in their approaches (Multimedia Appendix 1). Notably, 2 sites implemented practice-wide approaches with many clinicians (UC and UW), and 2 recruited volunteer clinicians (BIDMC and DHMC). Two sites (UC and UW) used Epic (Epic Systems Corporation, Verona, Wisconsin) to facilitate the insertion of patients’ submissions into notes. They were able to launch 9 months earlier than the other 2 sites.

In all sites, invitations to patients (Multimedia Appendix 2) included links to the form (Multimedia Appendix 3). The invitation described the intervention, explained that completing the form was voluntary, and noted that the form might not be read if they did not keep the appointment. At BIDMC, UC, and UW, automated processes sent the previsit invitations; physicians at DHMC selected patients from the weekly schedule, and clinic staff or the physician sent the invitation message.

### Evaluation

Pilot interventions were conducted between June 2018 and April 2020. Three sites (BIDMC, UC, and UW) used portal tracking data to monitor the use of the form throughout the pilot, and clinicians at DHMC made periodic estimates. The evaluation plan also included surveys of both clinicians and patients at each site and moderated discussions with the selected groups. However, the COVID-19 pandemic led to restrictions on research activities and diversion of information systems resources to support clinical care. Therefore, we were unable to complete all parts of the planned evaluation. We completed both patient and clinician surveys at the 2 sites that finished their 12-month pilots in 2019 (UC and UW), and surveyed clinicians at the other 2 sites (BIDMC and DHMC) in Spring 2020. However, without information systems resources to identify and track participants in early 2020, we were unable to conduct patient surveys at BIDMC or DHMC. We were also unable to convene focus groups.

The research team developed survey items based on pilot objectives, informal comments, and feedback from each site. We asked clinicians about the usefulness of patient responses, effects on workflow, and ideas for the future. We asked patients about the difficulties and benefits of answering the questions and about ideas for future improvements. To assess the burden of illness, we asked the patients if they had a chronic illness. The surveys included brief sets of sociodemographic items and both closed-ended and free-text questions. The questionnaires are available on request.

We invited clinicians who had received at least three completed previsit forms in the previous 6 months to complete the survey. We invited patients at UC and UW who had submitted at least one form during the pilot study. Clinicians in the 4 sites were contacted between June 2019 and July 2020, and UC and UW patients were contacted between September and October 2019. All participants received an invitation and up to two reminders. Each site offered an incentive of US $10 to US $50, following local guidelines. The surveys were conducted using Research Electronic Data Capture (REDCap, Vanderbilt).

### Analysis

Descriptive statistics were used to summarize response rates for the survey, survey responses, and the proportion of visits for which patients returned forms (using portal tracking data at BIDMC, UC, and UW, and clinician estimates at DHMC). In addition, patient results were stratified according to whether they reported having a chronic illness.

We completed thematic analyses of the free-text comments from the surveys. Patients were asked two questions: one for ways to improve the *OurNotes* previsit process, and one for other comments. To analyze responses, 2 authors first scanned the responses and noted that both questions drew comments related to both *OurNotes* and to other topics; therefore, we combined the comments for analysis. We reviewed 30 comments independently and drafted six topics, then independently reviewed 30 new comments and defined the final set of themes for coding: comments about the two questions, the form, workflow, patient engagement, whether the submitted form was read by clinicians, and others. Using REDCap, both authors coded all responses with up to three codes each, and 1 selected representative examples.

Clinicians were asked a single question about how to improve the previsit *OurNotes* process and any other comments. Two authors reviewed 30 comments to establish a list of nine themes, then 1 author reviewed all comments, assigned 1 or more themes to each, and selected representative thematic examples.

## Results

### Overview

In the 3 sites with portal tracking, patients were invited to complete forms before 58,652 scheduled visits, 5365 (9.1%) forms were submitted, and in the site without portal tracking, forms were returned before approximately 50% (260/520) visits (Multimedia Appendix 1). We invited 174 clinicians to complete the evaluation survey, and 76 (43.7%) responded. Two respondents did not remember receiving any completed forms, leaving an analytic data set of 74 clinicians. We contacted 1962 patients who had submitted at least one form and 525 (26.8%) responded; 204 did not remember completing a form, leaving an analytic data set of 321 patients.

### Clinician Results

Among the 74 clinician respondents, 49 (66%) were women, 61% (45/74) obtained their licenses since 2000, and 54% (40/74) reported 21 or more patient visits per week. On average, clinicians reported receiving between 1 and 3 patient forms per week in the previous 3 months (Table 1). Nearly all respondents agreed that having the information from the forms was a good idea, and 70% (52/74) reported that they usually or always incorporated responses of patients in the visit notes. Almost 90% (66/74) of clinicians reported that having the previsit information of patients either saved time or did not change the visit length.

**Table 1:** Responses from 74 clinicians about their experiences with OurNotes.^a^

Survey question	Response options	Value, n (%)
**In the last 3 months, about how many questionnaires did you receive from patients?**		
	<1/week	17 (23)
	1-3/week	38 (51)
	4-10/week	19 (26)
	>10/week	0 (0)
**In general, receiving the interim history before a visit is a good idea.**		
	Agree or somewhat agree	69 (93)
	Disagree or somewhat disagree	5 (7)
**In general, receiving the patient agenda before a visit is a good idea.**		
	Agree or somewhat agree	72 (97)
	Disagree or somewhat disagree	2 (3)
**How often did you incorporate some or all of what the patient wrote into your visit note?**		
	Never or rarely	12 (16)
	Sometimes	10 (14)
	Usually or always	52 (70)
**How having patients’ answers to the questions affected time spent writing notes?**		
	Took longer	4 (5)
	No change	61 (82)
	Took less time	9 (12)
**How useful were interim histories sent by patients?**		
	Not useful	7 (9)
	Somewhat useful	50 (68)
	Very useful	17 (23)
**How useful to have patients’ visit priorities at start of visit?**		
	Not useful	1 (1)
	Somewhat useful	31 (42)
	Very useful	38 (51)
	Not available at start	4 (5)
**Recall one or more instances where a patient’s submission was very important?**		
	Yes	25 (34)
	No	49 (66)
**Did it change the time to complete a patient encounter?**		
	Increased time	8 (11)
	No change	40 (54)
	Saved time	26 (35)

^a^Table excludes 2 respondents who did not remember receiving previsit forms.

The process of entering the responses of a patient to the record varied among the participating sites. In 2 sites where the previsit information could be automatically inserted into the visit note, 92% (45/48) preferred this approach. At the other 2 sites, most clinicians copied and pasted the information, 48% (11/23), read or typed all or part of the words of patients into the note, 13% (3/23), or simply referred to having read what the patient wrote, 30% (7/23).

Clinicians were asked to describe their experiences with the intervention and to suggest changes or improvements. Their 57 text responses centered on better notifications about completed forms, the need for easy insertion of patient text into the note, and ways to support patient participation (Textbox 1). Clinicians suggested that patients could be encouraged to be more engaged and taught about how to complete the form so that it would be more helpful for the visit.

**Examples from 57 clinicians’ responses to: “What changes/improvements would you suggest for the process tested in this pilot? Did it help or hinder you in providing care? Do you have other comments?”**

**Timely notifications to clinicians**

- Send the questionnaire results to my in-basket so I am more likely to see them before the visit.
- There were times I only learned of the patient's written comments from the patient during the visit. I think the patient was disappointed that I had not seen the comments in advance.

**Incorporating patient’s writing into note**

- It would be great if there was an easier way to incorporate patient notes into my own note.
- Improve the way the content of the message is incorporated into the body of the note: it would ideally blast in inside of quotation marks at whatever point the cursor is in the note body.

**Supporting patient participation**

- This could be very useful but for it to pay off would require “training ” my patients so that the responses are helpful to both patient and provider.
- Having this done on a regular basis would require a culture change where patients are less passive and more engaged and proactive about their care and ultimately get more out of each clinic visit.
- I do hope patients receive some type of disclaimer that their doctor might not be able to address all their concerns in a visit, even if the patient lists them all.
- Integration with our existing previsit questionnaires.

**Other**

- Good system. Please keep it very simple for patients and providers.
- [Customize for] differences between annual visits, [follow-up] visits, and urgent visits.

### Patient Results

Among the 321 patients who completed the survey from 2 sites (UC and UW), 57.9% (186/321) were women, 79.7% (255/320) were White, and 73.1% (234/320) had completed a 4-year college degree (Table 2). Half (158/320, 49.3%) of the respondents reported having three or more visits to their primary care provider in the previous 12 months, 68.2% (219/321) reported having one or more major chronic conditions, and 77.2% (247/320) reported their health as excellent, very good, or good. There were very few differences between respondents at the 2 sites (data not shown), other than more women responding in Colorado than in Washington (74% and 47% of respondents, respectively), and a higher proportion of respondents reporting White race in Colorado versus Washington (88% vs 74%, respectively).

**Table 2:** Self-reported characteristics of 321 patient respondents.^a^

Patient characteristic		Values, n (%)
**Age (years)**		
	18-44	78 (24.4)
	45-64	102 (31.9)
	≥65	140 (43.7)
**Sex**		
	Female	186 (58.1)
	Male	123 (38.4)
	Transgender	5 (1.6)
	Prefer not say	6 (1.9)
**Race**		
	White	255 (79.7)
	Black	11 (3.4)
	Asian, Hawaiian, or Pacific Islander	25 (7.8)
	Other	13 (4.1)
	Multiple races	16 (5.0)
**Ethnicity**		
	Non-Hispanic	300 (94.0)
	Hispanic	19 (6.0)
**Education**		
	Masters or doctoral degree	126 (39.4)
	4-year degree or some grad school	108 (33.7)
	Some college or technical school	72 (22.5)
	High school or less	14 (4.4)
**Overall health**		
	Excellent, very good, or good	247 (77.2)
	Fair or poor	73 (22.8)
**Chronic illness^b^**		
	Yes	219 (68.2)
	No	102 (31.8)
**Visits to primary care provider in last 12 months**		
	1-2	162 (50.6)
	3 or more	158 (49.4)

^a^Respondents from UCHealth University of Colorado Hospital and University of Washington Medicine only; we were unable to survey patients at Beth Israel Deaconess Medical Center and Dartmouth-Hitchcock Medical Center in Spring 2020 due to constraints imposed by the COVID-19 pandemic. Table excludes 204 respondents who did not remember completing any previsit forms.

^b^Do you have a chronic illness such as asthma, chronic obstructive pulmonary disease, diabetes, high blood pressure, arthritis, heart disease, or cancer?

More than half of the patients (170/321, 52.9%) completed more than one previsit form (Table 3). In general, few patients reported any difficulty, and more than 80.6% (259/321) had no difficulty answering the questions. About 68.8% (221/321) reported that answering the questions helped them prepare for the visit. Among the 85% (273/321) who reported reading their notes after the visit, 41% (112/273) found that the note included some or all that they had written on the previsit form, whereas more than one-third did not know whether what they had written was included in their visit notes. Patients overwhelmingly agreed that sending their commentary was a good idea (296/321, 92.2%). Most patients preferred to receive the previsit form 2-7 days before their visit. We found no substantive differences in responses between patients who did and did not report having a chronic illness (data not shown).

**Table 3:** Responses from 321 patients about their experiences with OurNotes.

Question	Responses	Value, n (%)
**In the last 12 months, how many times did you complete the OurNotes questionnaire before a visit?**		
	Once	151 (57)
	More than once	170 (53)
**How difficult was it to answer, “How have you been since your last visit?”**		
	Not at all difficult	259 (80.7)
	Somewhat difficult	60 (18.7)
	Very difficult	2 (0.6)
**How difficult was it to answer, “What issues would you like to focus on at your visit?”**		
	Not at all difficult	286 (89.1)
	Somewhat difficult	32 (10)
	Very difficult	3 (0.9)
**Did answering the questions help you prepare for the visit?**		
	Yes	221 (68.8)
	No	66 (20.6)
	Don’t know	34 (10.6)
**Did answering the questions change the conversation between you and your provider?**		
	Yes, positive effect	135 (42.1)
	No effect	100 (31.2)
	Yes, negative effect	7 (2.2)
	Don’t know	79 (24.6)
**Did answering the questions help you and your provider make decisions about your care?**		
	Yes, it helped	150 (46.7)
	No effect	90 (28.1)
	No, more difficult	3 (0.9)
	Don’t know	78 (24.3)
**After the visit, did you read your provider’s note?**		
	Yes	273 (85)
	No	33 (10.3)
	Don’t know	15 (4.7)
**Did the provider’s note include what you had written? (If yes)**		
	Yes, some or all	112 (41)
	No, but clearly provider had read my answers	35 (12.8)
	No, not included	23 (8.4)
	Don’t know	103 (37.7)
**In general, sending an update about myself before a visit is a good idea.**		
	Agree or somewhat agree	296 (92.2)
	Disagree or somewhat disagree	21 (6.5)
	Don’t know	4 (1.3)
**In general, sending the issues that I want to focus on before visit is a good idea.**		
	Agree or somewhat agree	301 (93.8)
	Disagree or somewhat disagree	17 (5.3)
	Don’t know	3 (0.9)
**When would you like to receive the request for your answers?**		
	2 days before the visit	131 (43)
	3-7 days before the visit	161 (53)
	>7 days before the visit	12 (4.0)

The patients wrote 282 comments in free text (Textbox 2). The coders agreed on codes for 161 responses and reached a consensus through discussion on the remaining 121. The comments most often (N=55) addressed the two questions in the form; about half wrote that they liked the questions as they were, and half found the questions too general. More specific comments related to the question asking for an interim history, with many making suggestions for more detailed queries, or requesting guidance on how to respond. The respondents wrote 33 comments about other aspects of the form and suggested some improvements. About half of the respondents commented on the space available for answers thought it adequate, and half wanted more room. Respondents saw the form as a tool supporting their engagement with care (27 comments), most often as a mechanism and incentive for collecting their thoughts before a visit. The 20 comments related to workflow often focused on improved visit efficiency, although several mentioned finding it inefficient when they were asked the same questions again at the visit; 27 comments referenced perceptions that the submitted forms were not read by their clinicians. Several suggested alerts to let the provider know the form was available, and to let the patient know it had been opened. Among other topics mentioned were concerns about privacy, patients for whom the form might present difficulties, and appreciation for specific staff, clinicians, portals, or institutions.

**Examples from 282 patient responses to: “What improvements would you suggest for the future?” and “Please write any other comments you would like to make.”**

**The 2 questions**

- The questions seem alright. It leaves room for the response to be personalized.
- The first question is a bit broad. It wasn't entirely clear to me how much detail my doctor would like or need.
- As someone not familiar with health care, I want more guidance around what is helpful for me to share with my provider about my health. I need examples or clearer descriptions.
- When it says “since last visit”—tell me when the last time I saw this provider was, I see several different doctors...

**The form**

- I think the form is easy to use, very clear and has just the right amount of space.
- I would not suggest making it any more complicated.
- The shorter the form, the more likely I'll do it.
- It would be helpful to have a brief recap of what our last visit entailed.
- Need more space and the ability to attach documents.

**Patient engagement**

- I like how it focuses me on what needs to be accomplished.
- Sometimes, I have to sit there and think about more than the first question, but that's ok.
- It leads me to the point that I have superior care in part because of my proactive participation in my own care.
- This program improves doctor-patient communication and facilitates my sense of being connected to the physician.
- I think leaving it so we can fill it in is super helpful. It makes some of the tougher subjects easier to approach.

**Workflow**

- I found that this makes the flow of the appointment better.
- I prefer to provide the updates online rather than filling out the form when I check in. It seems more efficient for all of us.
- Although, I spend time answering these, it seems I am asked again at the start of my appointment. This is repetitive and unnecessary.
- Some kind of follow up-did the provider open the note? After the visit, did my issues/concerns get addressed?
- Have the doctor have a printout before they enter patient’s room so they can discuss every issue together.

**Was the submitted form read?**

- I really don't know if it is read before the visit.
- The breakdown was from the doctor, who either had not read them or else for some reason wanted to quiz me to see if I knew what I had written...The time I had spent completing the notes was not treated with respect.
- I did not feel that the questionnaire was helpful, as it was not mentioned or referenced by the doctor or the team.
- I’m not sure the providers saw my answers before the visit. Perhaps they need an alert or flag when the patient has completed these questionnaires?
- It would be nice if I received some confirmation that my notes were received and read.

**Other**

- I would caution using this method with older people: my 79-year-old mother has difficulty with online medical forms and gets angry or frustrated with them.
- Too many complex medical issues for me to place this in a previsit note.
- I have some privacy issues with typing details of my doctor's concerns into the system in advance. I’m ok typing in the general purpose. I don’t have control over the information once I type it into the system and data tends to stick around forever.
- Let me know if it will be anonymous or who will know.

## Discussion

### Principal Findings

To our knowledge, this is the first study published on patients and clinicians cogenerating visit notes, or *OurNotes*. Patients were asked to prepare both interim histories about their conditions and their goals for an upcoming scheduled primary care visit, and participating patients and clinicians were strongly supportive. Consistent with our prime hypothesis, patients found completing the forms largely beneficial, and our hypothesis that *OurNotes* would have a neutral or positive impact on the time of clinicians appears to have been substantiated. We did not find additional benefits for patients with chronic illnesses.

In this pilot inquiry, participants offered many suggestions on how to improve the process. Patient respondents described how thinking through and providing the information supported their own engagement with care and helped visits flow more efficiently, but many wrote that they needed guidance about what to write. Clinicians often included the submissions of patients in their visit notes, suggested technical changes that would facilitate the process, and encouraged future efforts to teach patients to write interim histories that are more informative. Overall, both patients and clinicians suggest that this provides an important opportunity for better care. Based on this early proof of concept, all 4 sites decided after the 12-month pilot to continue the intervention, while considering also how to enhance this new approach to transparent patient-clinician communication and patient engagement.

### Challenges in the Pilot Implementation

Patients, in aggregate, submitted forms for fewer than 10% of eligible visits. We have no direct evidence from nonparticipants about why they chose not to send forms, and demographic data to analyze differences between them and participants were not available. A number of factors could explain this low level of participation, including insufficient communication with patients about the project before the intervention began, and the stated desire of patients in survey comments for guidance in answering the questions. The factors discussed below may have also contributed to this. Although evaluating reasons for nonparticipation was not among the objectives of this pilot study, it will be important to ascertain this in future research.

Although patients generally liked the idea of submitting information before visits, if such submissions are not readily available to providers before or during the visit, it could prove hurtful, as some patients may feel their input is being overlooked. Another challenge is how to support patients who may feel intimidated by being asked to write free text. Perhaps emphasizing the acceptability of writing phrases rather than complete sentences, allowing dictation functions such as those on smartphones, or offering other ways to record a verbal history may help. For patients to prepare and for clinicians to respond, designing a variety of effective approaches will require input from clinicians, patients, researchers, and information systems specialists.

Incorporating patient-written text into visit notes was an entirely new functionality in all 4 sites, and each designed and implemented an approach within the timeframe of the pilot to enable the most important steps in the process while attempting to minimize the impact on clinicians’ workflow. All sites experienced occasional glitches; clinicians commented particularly about sometimes not knowing that a patient had submitted a form until after the visit, and about issues with getting text of patients into their notes. After 12 months, each practice clearly required technical changes to facilitate various steps in the process. Although most clinicians reported no negative impact on their time, this important factor needs further study.

### Three Recommendations for Future Implementations

First, both patients and clinicians wanted a *closed communication loop* for previsit information. Clinicians expressed frustration about sometimes not knowing patients had submitted forms, or not being able to find them, and patients were disappointed or frustrated after taking time to complete the forms and then having their clinicians seemingly ignore what they had done. In a closed loop, clinicians would always know before an appointment that the patient had sent information, and patients would know whether the clinician had viewed their submissions. Using automated notifications for both patients and providers, technology can create such a system. Completed forms could be delivered directly to clinicians, although they worry about keeping up with an ever-growing in-box and potential medicolegal liability in unread items. Including links to submitted forms on daily schedules of clinicians could be one way to avoid additional messaging. Providers may also consider deadlines or messaging to encourage early patient submissions, in consideration for clinicians who review patients’ data the day or evening before a session, rather than at the time of the visit.

Second, both patients and clinicians explicitly asked for *education* that would help patients write *informative interim histories.* Some patients wrote of simply not knowing how to approach a request for a history. Lacking educational interventions, clinicians found the histories to be less useful than the visit priorities patients formulated. A foundational question concerns whether to ask patients to use a semistructured format, in addition to or in place of free text. In an informal meeting with members of one patient family advisory council, participants suggested that patients should be offered both options. Alternatively, might we prompt for interim histories by problem, or make forms that solicit unstructured histories before primary care visits, whereas those to subspecialists might be better suited to structured queries and responses?

Third, it is clear from the patient participation rate that we must learn more about how to *encourage patients* to take advantage of new opportunities to provide information to their clinicians outside of time-pressured encounters. For many, this involves registering more patients on patient portals, particularly those from vulnerable populations, and ensuring that they have adequate access, education, and support to use the portal [20,21]. Today, many patients fill out forms on paper or tablet computers in the waiting room [22]. Completing a web-based form at home facilitates timely review of prior open notes and may invite more consideration and thought, but it may also raise privacy concerns. Some survey respondents were intimidated by the form. Many of these concerns can be addressed with education, testimonials by patients and clinicians, and other outreach strategies. An appealing interface, dictation function, and seamless submission process may be helpful. Using some principles of behavioral economics, such as incentives for both patients and clinicians, may also improve rates of participation [23-25]. Encouraging patient engagement is always a multi-pronged, long-term undertaking.

### Limitations

This pilot study had important limitations. Most importantly, the proportion of patients who returned previsit forms was modest, and we were unable to conduct postimplementation evaluations with patients in 2 of the 4 sites because of the COVID-19 pandemic. Although we found no difference between the experiences of patients with and without chronic illness, we did not examine records to more fully ascertain their burden of illness. Response rates were within the usual range for web-based surveys [26], but nearly 40% of the patients did not remember submitting a previsit form, a function perhaps of having completed a single form many months before the survey, or of the ever-increasing amount of electronic communication with which patients contend. Finally, we did not collect quantitative information about the proportion of submitted forms that were actually used in the visits.

### Conclusions

Overall, in 4 different sites, each with a common goal but many degrees of freedom to design their own intervention, providers and patients liked the idea of patients providing information in advance of their visits and incorporating their observations into visit notes. Further development and research will be needed to assess barriers to patient participation, establish reliable notifications about information submitted by patients, and enable seamless incorporation of patient-generated text into notes.

This exploration is just beginning, and many questions arise. For both patients and clinicians, how should one balance time and effort before an encounter with time spent during a visit? Can cogenerating notes improve a crucial element of care: supporting mutual trust among patients, families, and clinicians? Could cogeneration of notes have particular benefits for those with multiple chronic conditions and those nearing the end of life? Can we effectively bring care partners into the process? Should we ask patients to think more broadly about their goals for health care, addressing issues germane to far more than an individual, time-constrained visit? As care delivery evolves in response to the COVID-19 pandemic, might *OurNotes* prove an effective component of telemedicine [27]? Future exploration may provide useful insights.

## Appendix

Multimedia Appendix 1 Implementation features of OurNotes in four sites.

Multimedia Appendix 2 Previsit invitation example.

Multimedia Appendix 3 OurNotes previsit form.

## Abbreviations

BIDMC: Beth Israel Deaconess Medical Center
DHMC: Dartmouth-Hitchcock Medical Center
REDCap: Research Electronic Data Capture
UCH: UCHealth University of Colorado Hospital
UW: University of Washington Medicine
