# Supplementary material for: Patients Contributing to Visit Notes: Mixed Methods Evaluation of OurNotes
Source: J Med Internet Res. 2021 Nov 8;23(11):e29951. doi: 10.2196/29951 (PMC8663611; doi:10.2196/29951)
Supplement: Multimedia Appendix 1 [file jmir_v23i11e29951_app1.docx]

**Multimedia Appendix 1. Implementation features of *OurNotes* in 4 sites**

|  | **UCH** | **UW** | **BIDMC** | **DHMC** |
| --- | --- | --- | --- | --- |
| Pilot dates | 6.12.18 – 6.30.19 | 6.1.18 – 6.30.19 | 2.25.19 – 4.20.20 | 4.1.19 – 3.30.20 |
| Participating clinicians | Primary care attending doctors and residents seeing participating patients in 2 primary care practices. | Primary care providers in 2 practices seeing participating patients: attending MD, fellow, resident, nurse practitioner, pharmacist, nutritionist, social worker. | 20 volunteering primary care doctors in a large hospital-based primary care practice. | 6 volunteering primary care doctors in 2 primary care practices. |
| Participating patients | Portal-registered patients with follow-up visits in 2 primary care offices; excluded initial, annual exam, procedure visits. | Portal-registered patients in 2 primary care practices with >3 visits in the UW system in the 12 months before the appointment. | Portal-registered patients on the panels of participating PCPs, minus 31 excluded by their PCPs. All visits except initial and scheduled <2 days before. | Patients selected from weekly schedule by participating PCPs. |
| How patients contacted before visits | Automated, included with pre-visit check-in 2 days before visit. | Automated email to patient with link to portal/form, 7 days before visit. | Automated portal message with link to form, 2 days before visit. | Primary care doctor or assistant sent 2 questions via portal message, patients replied via portal message. |
| Clinicians’  EHR workflow | Epic questionnaire. If doctor used standard progress note, Notewriter APSO auto-inserted patient responses into note. If not a standard note, doctor could use smart phrase to insert into note. | Epic questionnaire. Blue banner above notes field indicated patient form available, provider clicked “add” to insert or used smart phrase to attest to reading/verifying form. If note not added or attested to, provider reminded before closing encounter. | BIDMC EHR automatically stored form as a Patient Note pdf alongside provider notes, viewable by both doctor and patient. Doctor could mention viewing it, or copy/paste/type/dictate into note | Epic secure messaging, doctor could copy/paste into note. |
| # of visits with forms returned | 2,609 of 16,951 visits (15.4%) | 2,078 of 35,434 visits (5.9%) | 678 of 6,267 visits (10.8%) by 565 patients. | Actual counts not available. Estimate 260 visits (10 visits/week, 50% returned). |
| Contacted for evaluation survey | 59 doctors who received >3 forms, 33.9% response rate (RR).  1,098 patients who submitted >1 form, 21.5% RR. | 89 providers who received >3 forms, 33.7% RR.  862 patients who submitted >1 form, 39.6% RR. | 20 doctors who received >3 forms, 100% RR. | 6 participating doctors, 100% RR. |
|  |  |  |  |  |
