# Supplementary material for: Patients Contributing to Visit Notes: Mixed Methods Evaluation of OurNotes
Source: J Med Internet Res. 2021 Nov 8;23(11):e29951. doi: 10.2196/29951 (PMC8663611; doi:10.2196/29951)
Supplement: Multimedia Appendix 2 [file jmir_v23i11e29951_app2.docx]

**Multimedia Appendix 2. Pre-visit invitation example**

Subject: ***OurNotes*: Get the most out of your upcoming visit**

Dear Patient,

Welcome to *OurNotes*, a new project of the international [OpenNotes](https://www.opennotes.org/) movement created by patients and health professionals. For several years, BIDMC has been inviting you to read notes, and now *OurNotes* asks you to contribute important information that will become part of your electronic medical record. We believe *OurNotes* will improve care by getting patients, families, and clinicians even further onto “the same page.”

**You have a visit scheduled soon**. Your primary care physician has volunteered to join *OurNotes* and is asking you to **send some updates now**, before the visit. Your doctor will be able to view what you write before or during your office visit and may include your language in the note that s/he writes about the visit.

Please complete the pre-visit form and return it before your upcoming visit by clicking here:

<**form URL**>

Completing the pre-visit form is voluntary and your answers will be confidential. Your updates will become a permanent part of your record and viewable as a Patient Note in PatientSite. As with your entire record, they can be reviewed by other clinicians involved in your care. Please know that choosing not to complete the form will not affect your BIDMC care in any way.

If you have questions or concerns, or if you do not wish to participate in the *OurNotes* project, please send us a note at [opennotesresearch@bidmc.harvard.edu](mailto:MyOpenNotes@bidmc.harvard.edu) or contact the BIDMC Human Subject Protection Program at 617-667-0469.

Many thanks for joining OurNotes!

Sincerely,

**IMPORTANT**:

If you have an emergency or an urgent health issue, please do **not** use this form; instead, call 911 or go to the nearest emergency room.

Please understand that your doctor may not review your completed form until your visit (not before your visit). It may not be reviewed at all if you do not keep your appointment**.**
