# Supplementary material for: Patients Contributing to Visit Notes: Mixed Methods Evaluation of OurNotes
Source: J Med Internet Res. 2021 Nov 8;23(11):e29951. doi: 10.2196/29951 (PMC8663611; doi:10.2196/29951)
Supplement: Multimedia Appendix 3 [file jmir_v23i11e29951_app3.docx]

**Multimedia Appendix 3. *OurNotes* pre-visit form**

**How have you been since your last visit?**

For example: have you had any medication changes, new symptoms, or life changes? You may wish to read your provider’s last note and comment about what has happened since then.

*2,000 character limit (about 300 words)*

**What are the most important things you would like to discuss at your visit? (List up to 3)**

*300 character limit total (about 50 words)*

1.

2.

3.

Date/Time stamp added when submitted
